# Lunar Phases and Wildlife–Vehicle Collisions: Application of the Lunar Disk Percentage Method

**DOI:** 10.3390/ani11030908

**Published:** 2021-03-22

**Authors:** Gytautas Ignatavičius, Alius Ulevičius, Vaidotas Valskys, Lina Galinskaitė, Peter E. Busher, Giedrius Trakimas

**Affiliations:** 1 Institute of Biosciences, Life Sciences Center, Vilnius University, 10257 Vilnius, Lithuania; alius.ulevicius@gf.vu.lt (A.U.); vaidotas.valskys@gmc.vu.lt (V.V.); lina.galinskaite@gmc.stud.vu.lt (L.G.); giedrius.trakimas@gf.vu.lt (G.T.); 2 College of General Studies, Boston University, Boston, MA 02215, USA; pbusher@bu.edu

**Keywords:** wildlife–vehicle collisions (WVCs), lunar phases, lunar disk percentage (LDP), behavior

## Abstract

**Simple Summary:**

The moon is ubiquitous in the night sky and considered an important abiotic factor that influences animal activity. However, little is known about the relationship between moonlight and the daily, monthly, or seasonal frequency of wildlife–vehicle collisions (WVCs). Traditionally, the influence of moonlight on WVCs has been analyzed using the lunar phase (quarters) approach, which evaluates moonlight on a rough scale (only four 25% steps of the visible moon disc and a strict arrangement of phases over time). We used a different approach; we compared WVCs to the actual lunar disc illumination that is based on the specific daily percentage of the visible lunar disk (LDP). Our findings indicated a significant trend of increasing WVC frequencies with increasing LDP at night. We also examined the correlation between the daily numbers of WVCs and LDP for different months and seasons. Positive correlations between LDP and WVCs were stronger at night and during the late autumn–winter months, particularly in December, suggesting the importance of lunar illumination on WVCs. Our study suggests that the LDP approach may provide more possibilities for the evaluation and quantification of WVCs and lunar light relationships than the traditional lunar phase approach. The results can be useful for predicting and reducing WVCs at different times of the lunar illumination cycle and in different seasons.

**Abstract:**

We investigated the relationship between lunar illumination based on the percentage of the visible lunar disk (LDP) and the frequency of wildlife–vehicle collisions (WVCs) in Lithuania. We analyzed WVC frequency during ten 10% LDP intervals to more precisely reflect the relationship between LDP and WVC. The 10% LDP interval approach showed a significant trend of increasing WVC frequencies with an increasing LDP at night. We also examined the correlation between the daily numbers of WVCs and LDP for different months and seasons. The relationship seemed to be stronger at night and during the late autumn–winter months, particularly in December, suggesting the importance of lunar illumination on WVCs. There was a weak positive correlation between LDP and overall daily number of WVCs (r_s_ = 0.091; *p* < 0.001) and between LDP and night WVCs (r_s_ = 0.104; *p* < 0.001). We found significant positive correlations for winter (December–February) (r_s_ = 0.118; *p* = 0.012) and autumn (August–November) (r_s_ = 0.127; *p* = 0.007). Our study suggests that the LDP interval approach may provide more possibilities for the evaluation and quantification of WVCs and lunar light relationships than the traditional lunar phase approach.

## 1. Introduction

Increasing urbanization and the subsequent construction and increased use of roadways (both large and small) have increased the probability of human–wildlife interactions that are costly for all species involved [1,2]. Wildlife–vehicle collisions (WVCs) continue to be a serious problem for both humans and wildlife species near urban areas, at the wildland–urban interface, and along all roadways [3,4,5]. For example, Forman and Alexander [6] estimated that over a million vertebrates are killed per day in the United States. In Europe, more than 500,000 collisions with ungulates occur annually, resulting in approximately 30,000 injuries and an economic cost of $1 billion [7]. In Lithuania, where our study was conducted, insurance company data indicated that the average economic loss due to WVCs was 1500 euros and that, in some cases, the economic loss exceeded 24,000 euros. The estimated total economic loss per year in Lithuania due to over 4000 annual WVCs is approximately 6,000,000 euros. However, this amount does not include losses incurred due to injuries and deaths, which would significantly increase the annual cost of WVCs. Every year, more than 100 people are injured and at least one person is killed due to WVCs [4]. Many factors that influence the frequency of WVCs have been investigated, including biotic factors (wildlife species density, diel and seasonal behavior, roadside vegetation, and human traffic behavior) and abiotic factors (topography, weather, time of day, and season) [8,9,10,11,12,13]. Many studies have reported temporal patterns in WVCs, and the most frequently mentioned reasons for this temporal pattern are the reduced ability of drivers to react in time to wildlife on the road due to visibility constraints and the crepuscular and nocturnal activity of animals [14,15,16,17]. While WVCs have been documented to occur at all times of the day (24 h period), the majority of WVCs with large animals (primarily ungulates) occur at night [18,19,20].

The moon is an ever-present feature in the night sky, and its illumination changes during the monthly lunar cycle. Numerous studies have documented the relationship between the monthly lunar cycle (and illumination) and the activity of various animal species [21,22,23,24,25]. The primary influence of moonlight on wildlife species is on their movement patterns associated with foraging and/or predation risks. Increased lunar illumination makes both predator and prey more visible, and it can either increase or decrease movement [26,27]. Thus, increased moonlight may be a fundamental factor that influences animal movement patterns during the monthly lunar cycle. Large mammals may change their crepuscular and/or nocturnal activity patterns under changing lunar illumination with consequent changes in their involvement in WVCs.

However, despite being recognized as an important abiotic factor that influences animal activity, the lunar cycle has received little attention as a potential variable in WVCs [12,28,29,30]. It has been reported that some wildlife species are more likely to be involved in WVCs during the full moon phase [28,31,32], but the influence of the moon on WVCs during all stages of illumination needs more study. A better understanding of how the specific lunar phase or the percentage of the lunar disc that is illuminated correlates with WVCs can significantly contribute to predicting and reducing the number, consequences, and cost of WVCs. Most researchers analyzing the correlation between moonlight and WVCs have relied on an assessment of the influence of the different lunar phases. For example, Colino-Rabanal et al. [28] correlated WVCs for four species on two continents with the four primary lunar phases (full, third quarter, new, and first quarter). While providing valuable information on the relationship between WVCs and lunar phase, this approach may underrepresent the importance of lunar illumination during the entire lunar illumination cycle (0–100%). By only correlating WVCs with specific phases, you are only getting four glimpses of how lunar illumination is correlated with WVCs. This methodological shortcoming can be overcome by relating lunar disk illumination to the percentage of lunar disk visible during the lunar cycle and using the precise lunar disk percentage (LDP) to analyze the frequency of WVCs. In this approach, all WVCs are attributed to exact LDPs throughout the lunar cycle, thus allowing for greater accuracy when examining the influence of lunar illumination on WVCs.

Our study was designed to compare the lunar phase method (four broad lunar phases/quarters—waxing crescent, waxing gibbous, waning gibbous, and waning crescent) with the lunar disk percentage method (0–100% disk visibility) in examining the influence of lunar illumination on WVCs in Lithuania. We also examined the correlation between the daily numbers of WVCs and LDP for different months and seasons. Our general question was: how do precise lunar disk percentages correlate with WVCs and how do they compare to WVC correlations with lunar phases? We used these methods to test the following hypotheses involving WVCs and lunar illumination.

1. The monthly cycle of lunar illumination is positively correlated with WVCs. Specifically, that increased lunar illumination is positively correlated with increased WVCs, which is most likely due to increased animal movement that increases the potential for collisions.

2. The seasonal lunar pattern (brighter during late autumn and winter compared to spring and summer) is positively correlated with increased WVCs. If accepted, this hypothesis suggests that animal movement is greater during parts of the year when lunar illumination is also greater and leads to a higher potential for WVCs at these times.

## 2. Materials and Methods

Records of all WVC accidents in Lithuania from 2014 to 2018 (14,437 records) were collected from the Lithuanian Road Police (LRP) Database. All WVC accident records are anonymous and are categorized by location with coordinates, time of the accident, the animal species involved, the specific circumstances, persons injured or killed, and other data. We analyzed all WVC events recorded on Lithuanian roadways for this study regardless of the wildlife species. We then analyzed the frequency distribution of WVCs compared to both the actual percentage of the lunar disc illuminated for both day (sunrise to sunset: 25.23% of WVCs) and night periods (sunset to sunrise: 74.77% of WVCs), as well as the lunar phase. The Scientific Visualization Studio (SVS) interactive program on the NASA website (https://svs.gsfc.nasa.gov/; accessed on 21 December 2020) was used to determine the lunar phase for each specific day. This program, at the request of the user, provides data on the percentage of the lunar disk illuminated, the distance of the Moon from the Earth, and other Earth–Moon information. Data for each day are presented on a separate webpage, and the SVS does not provide structured or selected data sequences. Thus, we used UiPath for task robotics and webscrape technology. We created an additional program that modified the SVS sheets and allowed us to select only information on lunar disk percentage for each day of 2014–2018 (provided in the xlsx format). We assigned the actual LDP for each day of 2014–2018 based on the NASA data and used the LDP in three ways.

First, we divided the monthly LDP cycle into 10 equal 10% intervals from 0% to 100% LDP, and then we assigned each day to an interval based on LDP. We then analyzed the number of WVCs on the days in each 10% interval. To standardize the results, WVCs observed in each interval were divided by the number of days in that interval and further analyzed by the number of collisions per day, with an additional separation of day and night collisions.

Second, for comparison, we also examined the correlation between WVCs and lunar phases. The assessment of the lunar phase was based on the standard astronomical assumption that the LDP of the new moon is 0%, the waxing crescent moon (WXC) is from 0.1% to 49.9%, the first quarter moon is 50%, the waxing gibbous moon (WXG) is from 50.1% to 99.9%, the full moon is 100%, the waning gibbous moon (WNG) is from 99.9% to 50.1%, the third quarter moon is 50%, and the waning crescent moon (WNC) is from 49.9% to 0.1%. We assumed that the titles new moon, first quarter moon, full moon and third quarter moon just indicate specific points but not the intervals of LDP; thus, in our analysis, the meaning “lunar phase“ indicates one of four intervals/quarters of LDP with a respective title (waxing crescent, waxing gibbous, waning gibbous, and waning crescent).

Third, we paired the number of WVCs and LDP with day and night periods to calculate correlations between these two indicators in different months and seasons (the continuous LDP data method). The duration of night time varies from 7:14 during summer to 16:46 during winter season. These seasonal differences are clearly expressed in higher latitudes but less pronounced in Mediterranean countries.

The number of wildlife–vehicle collisions per day during different lunar phases was compared using the Mann–Whitney U test. Comparisons between the dark (night) and light (day) time periods at different lunar phases were made using Wilcoxon signed rank test. We reported raw *p* values for these tests since all *p* values remained significant after corrections for multiple comparisons using Holm–Bonferroni method. Trend lines for dark (night) and light (day) periods of the 10% LDP interval data (mean WVCs per day) were assessed by Spearman’s rank-order correlations. We also ran Spearman’s rank-order correlations to determine the relationship between daily lunar disk percentage (continuous variable) and the daily number of WVCs. Lastly, we tested for seasonal (winter, spring, summer, and autumn) correlations between overall daily WVCs and LDPs. All tests were two-tailed if not stated otherwise.

## 3. Results

An analysis of the database revealed that 63.2% of all WVCs involved roe deer (*Capreolus capreolus*), 6.9% involved moose (*Alces alces*), 4.7% involved wild boar (*Sus scrofa*), 1.4% involved red deer (*Cervus elaphus*), and 0.1% involved fallow deer (*Dama dama*). Non-identified wildlife and domestic animals comprised the remaining 23.7% of WVCs.

**Figure 1:**
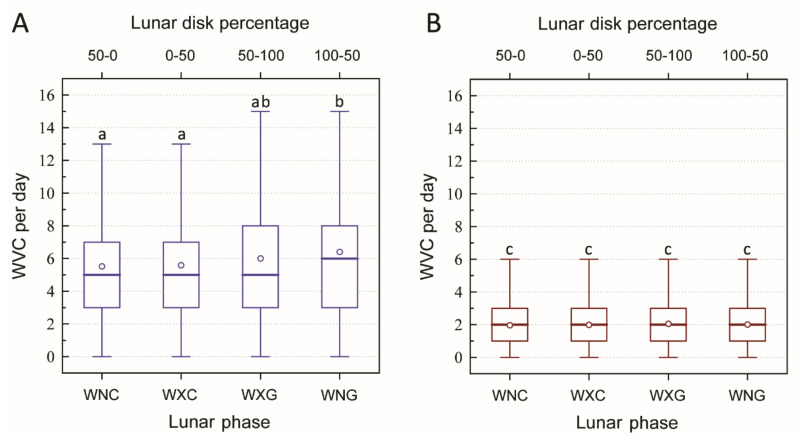
Wildlife–vehicle collisions (WVCs) per day during different lunar phases. (**A**) = dark period (night—sunset to sunrise); (**B**) = light period (day—sunrise to sunset). Lunar phases are arranged by the percentage of lunar disc: waning crescent (WNC), waxing crescent (WXC), waxing gibbous (WXG), and waning gibbous (WNG). Thick lines indicate medians, open circles indicate means, boxes indicate inter quartile ranges, and whiskers indicate non-outlier ranges. Lowercase letters (above the whiskers) indicate statistical significance (Mann–Whitney U test (*p* < 0.05))—phases with the same letter are not statistically different from each other, while phases with different letters are statistically significant from each other.

At night (dark period—sunset to sunrise), there was a tendency for more WVCs per day during the waxing gibbous (mean and 25th–75th percentiles: 6.04 and 3–8, respectively; n = 455) and waning gibbous (mean and 25th–75th percentiles: 6.41 and 3–8, respectively; n = 468) phases than during the waning crescent (mean and 25th–75th percentiles: 5.55 and 3–7, respectively; n = 455) and waxing crescent (mean and 25th–75th percentiles: 5.62 and 3–7, respectively; n = 450) phases (Figure 1A). The number of WVCs per day during the waning gibbous phase was significantly higher than during the waning crescent and waxing crescent phases (Mann–Whitney U tests: *p* < 0.001 and *p* < 0.004, respectively), but not significantly different during the waxing gibbous phase (Mann–Whitney U test,: *p* = 0.107) (Figure 1A). There were significantly fewer WVCs per day during the day (light period—sunrise to sunset) in comparison to the night (Wilcoxon signed-rank tests: all *p* < 0.001). We found no significant differences in WVCs per day between the lunar phases during the day (light period) phases of waning crescent (mean and 25th–75th percentiles: 1.96 and 1–3; respectively; n = 455), waxing crescent (mean and 25th–75th percentiles: 1.98 and 1–3, respectively; n = 450), waxing gibbous (mean and 25th–75th percentiles: 2.03 and 1–3, respectively; n = 453), and waning gibbous (mean and 25th–75th percentiles: 2.0 and 1–3, respectively; n = 468) (Mann–Whitney U test: all *p* > 0.05) (Figure 1B).

**Figure 2:**
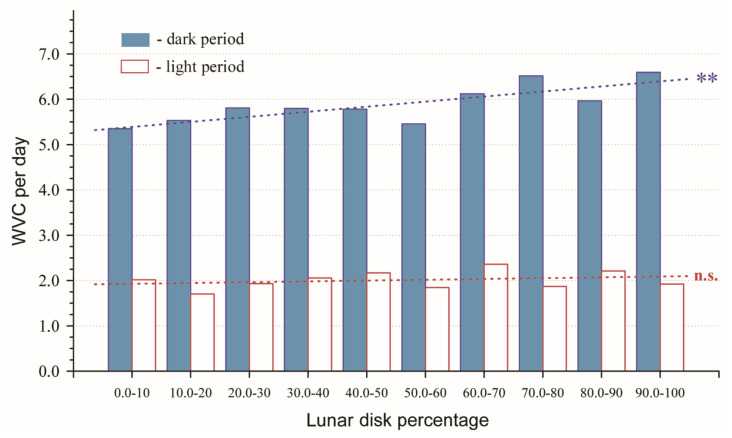
Average wildlife–vehicle collisions (WVCs) per day for 10% interval lunar disk percentages. Filled columns = dark period (night—sunset to sunrise); open columns = light period (day—sunrise to sunset). Asterisks indicate significant trendline (r_s_ = 0.79; *p* = 0.006). n.s. = non-significant.

There was a significant positive correlation between ten ordered LDP ranks and the mean WVC number that occurred during the dark period (r_s_ = 0.79; *p* = 0.006; n = 10), indicating increasing WVCs with increasing LDPs (Figure 2). During the light period, we found no significant (positive or negative) correlation between WVCs and LDP (r_s_ = 0.19; *p* = 0.60; n = 10) (Figure 2).

The continuous LDP data method also revealed a statistically significant weak positive correlation between the total daily (24 h period) number of WVCs and LDP interval (r_s_ = 0.091; *p* < 0.001; n = 1826). There was a significant positive correlation between LDP and the number of WVCs that occurred during the dark period (r_s_ = 0.104; *p* < 0.001; n = 1826) but no significant correlation between the LDPs and WVCs that occurred during the light period (r_s_ = 0.002; *p* = 0.944; n = 1826).

We observed significant positive correlations between the LDP and the total daily number of WVCs during winter (December–February) (r_s_ = 0.118; *p* = 0.012; n = 451) and autumn (August–November) (r_s_ = 0.127; *p* = 0.007; n = 456) but no significant relationship between the LDP and the total daily number of WVCs during summer (r_s_ = 0.089; *p* = 0.055; n = 461) and spring (March–May) (r_s_ = 0.05; *p* = 0.288; n = 460). There were significant relationships between WVCs that occurred during the dark period and the LDP for winter (r_s_ = 0.145; *p* = 0.002; n = 451), summer (r_s_ = 0.11; *p* = 0.018; n = 461), and autumn (r_s_ = 0.134; *p* = 0.004; n = 456), but no significant relationship was observed for spring (r_s_ = 0.053; *p* = 0.256; n = 460). We observed no significant correlations between WVCs that occurred during the light period and the LDP in all seasons (*p* > 0.05).

**Figure 3:**
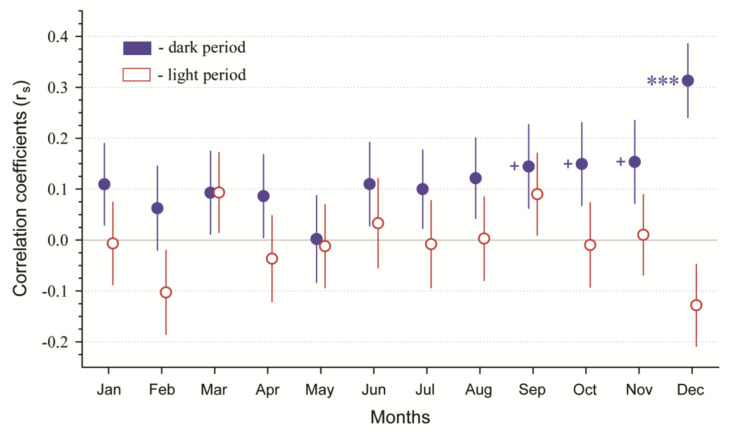
Spearman’s correlation coefficients between wildlife–vehicle collisions (WVCs) and lunar disk percentages (LDPs) during 2014–2018 in Lithuania. Closed circles indicate correlation coefficients between WVCs and LPD that occurred during the dark period (night), while open circles indicate correlation coefficients between WVCs and LDP during the light period (day). Whiskers indicate Std. errors based on 10,000 bootstrap replicates. *** indicates *p* < 0.001, and + indicates one-tailed significance; *p* < 0.05.

The analysis of the monthly relationships between the WVCs that occurred during the light period (day WVCs) or the dark period (night WVCs) and the LDP indicated the tendency for correlation coefficients to be higher between the night WVCs and LDP than the day WVCs and LDP. However, the only significant correlation between the night WVCs and LDP was found for December (r_s_ = 0.313; *p* < 0.001; n = 155), with marginally non-significant correlations observed for September (r_s_ = 0.144; *p* = 0.078; n = 150), October (r_s_ = 0.149; *p* = 0.064; n = 155), and November (r_s_ = 0.154; *p* = 0.061; n = 150). Correlations between the night WVCs and LDP for these months would be significant if using a one-tailed test. All other correlations (light period and dark period) were non-significant (*p* > 0.05) (Figure 3).

## 4. Discussion

Our data support the findings of other researchers that WVCs are more apt to occur during periods of higher illumination such as near the full moon phase [28,31,32]. Both (lunar phase and LDP) methods allowed us to accept our first hypothesis that increasing lunar illumination, expressed by an increasing visible lunar disk percentage, was correlated with a higher number of WVCs, though trend lines were rather flat and correlation coefficients were low. In other regions, significantly more expressed differences in WVC frequencies between the new and full moon conditions were found. For example, in Spain, WVCs were 71.3% greater during the full than new moon period [28]. This discrepancy in the quantitative response of WVCs vs moon light could be explained by differences in cloud coverage (number of days, percentage, etc.) between Lithuania and Spain.

A significant positive correlation between WVCs and LDP during the night for autumn and winter but not for spring and summer generally supported our second hypothesis that WVCs show a seasonal pattern that may be related to greater importance of lunar illumination when nights are getting longer. December, the month with the longest nights in the year, was the only month with the highest correlation between WVCs and LDP. The duration of night might be one of the factors that shapes the WVC and LDP relationship. Possible explanations include the higher needs of animals to search for food during long and bright nights and changes of resting sites. The duration of night in Lithuania varies from 7 h 14 min in June to 16 h 46 min in December. These seasonal differences are clearly expressed in higher latitudes, which is not so characteristic elsewhere, e.g., in the Mediterranean region.

While our analysis examined all species of wildlife involved in vehicle collisions, the majority of WVCs in Lithuania involved roe deer (over 63%). Roe deer behavior is thought to have the highest impact on roe deer–vehicle collisions (RDVCs) [12,20]. Roe deer have a 24-h activity cycle that consists of 8–12 activity bouts (polyphase activity), with the shorter bouts observed at night [33]. Wallach et al. [34] reported a significant increase in activity during colder months (winter). However, nocturnal activity was reported to be greater in summer than in winter when activity peaks became more crepuscular and deer were more active near dawn and dusk [35]. Our earlier investigations of RDVCs in Lithuania found five months (May, October, November, December, and January) to have the highest number of collisions. However, it is interesting that December, the month in which the WVCs and LDP were most closely correlated in this present study, was not the peak month for collisions. Daily (24-h) RDVC distribution in late autumn and winter was clearly bimodal and almost evenly distributed between morning and evening hours. However, in summer, RDVCs were clearly associated with evening hours [4].

Lunar illumination may influence roe deer feeding behavior by extending the length of browsing/grazing bouts during the long nights in late autumn and winter. On dark, moonless nights, deer movement may be suppressed, possibly to reduce predation risk. At dawn and dusk and on nights with greater lunar illumination, predation risk is decreased and deer can more actively search for food. Additionally, D‘Angelo et al. [36] suggests that roe deer crepuscular vision specialization also supports effective browsing during nights with increased lunar illumination.

The need for increased food consumption during colder seasons also could force roe deer to extend their nocturnal feeding time that would be enhanced by greater lunar illumination. Additionally, higher energy requirements due to heat loss in cold and wet environments may require longer foraging periods and/or extend the amount of time spent in foraging activities. For example, Siberian roe deer in the Southern Ural area were found to have a significantly higher food consumption in winter than in summer [37].

Roe deer feeding behavior is characterized by slow but constant directional motion (animals do not remain feeding in one spot) when browsing or grazing [33]. Only when there is a very high concentration of food will a deer rest for the night where they forage (e.g., in abandoned gardens or close to human settlements). In mild winters (like the last several winters in Lithuania), roe deer forage more as grazers than browsers due the higher nutritional value of grass compared to twigs [38]. In winter, when grass is more sparsely distributed over large areas, roe deer must spend additional time searching for food to meet their nutritional requirements.

These general patterns of roe deer behavior may contribute to increasing RDVCs during nights with more lunar illumination. However, we are well-aware that many other factors also can influence this relationship. The weak correlations we observed might have been caused by other meteorological circumstances. For example, it was impossible to reconstruct the actual amount of cloud cover and precipitation for every collision case that could have influenced the total lunar illumination. Nonetheless, Stubbe [39] reported that roe deer decrease their activity in bad weather conditions and during dark nights (when lunar illumination is limited). This suggests that the relationship between greater lunar illumination and increased roe deer movement is real and is a factor in WVCs.

Understanding the relationship between lunar illumination (lunar phases) and animal collisions with vehicles must consider both animal and driver behavior. Our data suggest that more lunar illumination influences animal movement, bringing them in greater contact with roadways, perhaps more than it enhances the ability of drivers to see wildlife and avoid collisions. Many authors have reported on the influence of lunar phases on the number of vehicle accidents, thus indicating the importance of human behavior [17,40,41]. Animal and human behaviors can have an additive effect on WVC patterns at night, especially seasonally when days get shorter and nights longer. However, this relationship needs to be more deeply investigated to fully understand its importance to WVC causality. The general thought is that human behavior and activity is somehow related with lunar phases [42].

In this study, we used a new approach (lunar disk percentage method) to examine the relationship between lunar illumination and WVCs compared to the lunar phase method. Our results indicated both similarities and subtle but significant differences between the lunar phase method and the LDP method in analyzing the influence of lunar illumination on WVCs. Our LDP method (ten 10% intervals) allowed for a more sensitive analysis of the effects of lunar disk illumination on WVCs than the rough-scale lunar phase method, which essentially only compares two broad periods of lunar disc percentage periods (50–0–50% and 50–100–50%). The use of the lunar phase method may miss subtle patterns of how wildlife responds to lunar disk illumination and its influence on WVCs. For example, both of our methods indicated that increasing illumination at night correlated to a higher number of WVCs. However, our lunar phase method missed potentially important incremental changes in illumination. For example, our LDP method, while indicating an increasing trend with increasing illumination, found a similar number of WVCs during 0–10% illumination and 50–60% illumination. It is also interesting that the number of WVCs reported during times of 70–80% illumination were almost the same as during 90–100% illumination. Consequently, the LDP method should lead to more reliable predictions and solutions. While our data showed a continuous tendency of WVCs and lunar illumination, we do not exclude that there may be a threshold component to lunar illumination vs WVC dynamics.

Further studies examining the role of lunar illumination on WVCs should include weather conditions, especially cloud cover and precipitation (which we were unable to include). Additionally, it would be interesting to examine non-wildlife accidents (human vehicle accidents) and lunar illumination using our LDP method. Our results add to the existing knowledge of how lunar illumination and WVCs are related and can be used in predicting and reducing WVCs by defining daily, monthly, and seasonal high risk periods.

## 5. Conclusions

1. The results of our analysis allow us to accept our first hypothesis that increasing lunar illumination, expressed by increasing the percentage of the visible lunar disk, is correlated with a higher number of WVCs, though trend lines were rather flat and correlation coefficients were low, probably due to the specificity of weather conditions (e.g., high cloudiness) in Lithuania.
2. Wildlife–vehicle collisions and LDP were significantly correlated during the dark period (night) during winter, summer, and autumn but not spring. No seasonal influence was observed for the light period (day) between WVCs and LDP. We also observed a general trend of increasing correlation between WVCs and LDP by month, especially from June through December. December was the only month in which the correlation between WVCs and LDP during the dark period was highly significantly different from the other months.
3. Our LDP method (ten 10% intervals of the visible lunar disc percentage) allowed for a more refined analysis of the effects of moonlight on WVCs than the broader-scale lunar phase method, which essentially only compares two broad periods of lunar disc percentage periods (50–0–50% and 50–100–50%). Specifically, the LDP method allowed for a more sensitive analysis monthly and seasonal patterns between lunar illumination and WVCs.
